# A Rare Case of Traumatic Internal Mammary Artery Pseudoaneurysm Causing Hemopericardium: Endovascular Management and Literature Review

**DOI:** 10.15388/Amed.2025.32.2.16

**Published:** 2025-12-30

**Authors:** Shritik Devkota, Rajan Mani Bhandari, Jasmine Sethi, Harish Bhujade

**Affiliations:** 1 Department of Radiodiagnosis and Imaging, Postgraduate Institute of Medical Education & Research (PGIMER), Chandigarh, India; 2 Department of Radiodiagnosis and Imaging, Postgraduate Institute of Medical Education & Research (PGIMER), Chandigarh, India; 3 Department of Nephrology, Postgraduate Institute of Medical Education & Research (PGIMER), Chandigarh, India; 4 Department of Radiodiagnosis and Imaging, Postgraduate Institute of Medical Education & Research (PGIMER), Chandigarh, India

**Keywords:** internal mammary artery pseudoaneurysm, traumatic IMA pseudoaneurysm, endovascular embolization, vidinė krūties arterijos pseudoaneurizma, trauminė IMA pseudoaneurizma, endovaskulinė embolizacija

## Abstract

**Background:**

Traumatic *Internal Mammary Artery* (IMA) pseudoaneurysms can be associated with severe complications, including hemopericardium, pericardial tamponade, diaphragmatic paralysis, hemoptysis, and hemorrhagic shock. Early recognition of this rare condition is essential to reduce the risk of significant morbidity and mortality.

**Case details:**

We present a case of IMA pseudoaneurysm that was promptly diagnosed by using contrast-enhanced CT and successfully managed with endovascular embolization, leading to the patient’s complete and uneventful recovery.

**Conclusion:**

This case emphasizes the importance of early recognition and endovascular management of rare but potentially life-threatening internal mammary artery pseudoaneurysms following thoracic trauma.

## Abstract (it)

**Įvadas:**

Trauminės vidinės krūties arterijos (IMA) pseudoaneurizmos gali sukelti sunkių komplikacijų, įskaitant hemoperikardą, perikardo tamponadą, diafragmos paralyžių, hemoptizę ir hemoraginį šoką. Kuo anksčiau atpažinti šią retą būklę yra labai svarbu, siekiant sumažinti didelę sergamumo ir mirtingumo riziką.

**Atvejis:**

Pateikiame IMA pseudoaneurizmos atvejį, kuris buvo greitai diagnozuotas naudojant kontrastą sustiprinančią kompiuterinę tomografiją ir sėkmingai išgydytas atlikus endovaskulinę embolizaciją, dėl kurios pacientas visiškai ir be komplikacijų pasveiko.

**Išvados:**

Šis atvejis pabrėžia ankstyvojo atpažinimo ir endovaskulinio gydymo svarbą retų, bet potencialiai gyvybei pavojingų vidinių krūties arterijos pseudoaneurizmų po krūtinės ląstos traumos atvejų.

## Background

Cardiothoracic trauma, whether blunt or penetrating, often results in a variety of injuries, including rib fractures, hemothorax, hemopericardium, pulmonary lacerations, cardiac or pulmonary contusions, with vascular complications like arterial pseudoaneurysms being a recognized sequela.[1,2] These pseudoaneurysms are abnormal outpouchings of the arterial wall, typically contained by surrounding hematoma. Although rare, *Internal Mammary Artery* (IMA) pseudoaneurysms can occur following trauma. This case report describes a 34-year-old male who sustained a sharp nail injury resulting in traumatic hemopericardium with tamponade and an IMA pseudoaneurysm, which was diagnosed on contrast-enhanced CT (CECT) and successfully treated with endovascular embolization. This highlights the effectiveness of endovascular approaches in managing such complex vascular injuries.

## Case Presentation

### Clinical History and Investigations

A 34-year-old male carpenter presented to the emergency department of a tertiary care hospital about 30 minutes after sustaining a sharp nail injury to the chest at work. The nail had been removed by his companion at work place. There was a pinpoint entry wound with focal skin irregularity which was located on the left anterior chest wall (parasternal) just approximately 2 cm beneath the nipple line. It measured approximately 10 mm in diameter. There was no swelling, skin changes, or active bleeding.

The patient reported sharp pain in the anterior chest wall and shortness of breath after removal of the nail. On examination, he was tachycardic, with a heart rate of 125 beats per minute. He was tachypneic, breathing 31 times per minute. His blood pressure was low at 100/60 mmHg. Oxygen saturation was 91% on room air. Intravenous access was secured. He was given a bolus of IV fluids and supplemental oxygen immediately. Blood was arranged without delay.

A portable extended focused assessment with sonography in trauma (eFAST) showed hemopericardium with cardiac tamponade and a left-sided hemothorax. The chest radiograph revealed no pneumothorax or rib fractures. Initial blood tests showed a low hemoglobin level of 7.5 g/dL.

**Figure 1:**
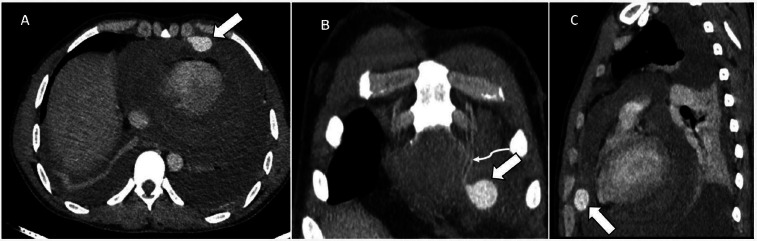
Axial (A), coronal (B) and sagittal (C) sections of CECT chest showing bilateral hemothorax, hemopericardium and contrast filled outpouching (the white arrow) arising from the left internal mammary artery (the curved arrow)

The patient was quickly transferred to the radiology department for a CECT of the chest. During the transfer, he received one unit of whole blood. The CECT showed a contrast-filled outpouching from the left IMA. It measured about 1.5 × 2.1 × 2.2 cm (anteroposterior × transverse × craniocaudal). This was consistent with a pseudoaneurysm. In addition, there was hemopericardium, and hemothorax. No active contrast extravasation or pulmonary parenchymal injury was noted (Figure 1).

The diagnosis of hemopericardium by eFAST was made within approximately 15 minutes after the patient’s presentation to the hospital, and the diagnosis of IMA pseudoaneurysm with hemopericardium via CECT was established within approximately 38 minutes of presentation (i.e., 23 minutes from eFAST to CT diagnosis).

Cardiac injury was initially a significant concern given the clinical presentation. However, the presence of a pseudoaneurysm with adjacent hemopericardium, along with the superficial nature of the injury and normal cardiac motion observed following pericardiocentesis aspiration, suggested that direct cardiac injury was less likely.

### Management and Outcome

After stabilizing the patient’s vitals with two more units of blood transfusion and supplemental oxygen, ultrasound-guided needle aspiration of the hemopericardium was performed to relieve cardiac tamponade. Since there was no active contrast extravasation on imaging, no catheter drainage was done. The cardiology team remained on standby for immediate intervention if the hemopericardium expanded or required further drainage. Following a multidisciplinary discussion involving the emergency physician, cardiologist, interventional radiologist, and cardiothoracic surgeon, the patient was referred for endovascular embolization. The cardiothoracic surgery team was kept on standby to perform thoracotomy if embolization failed to relieve the patient’s symptoms. The treatment plan, including risks and benefits, was thoroughly explained to the patient and family, and informed consent was obtained.

**Figure 2:**
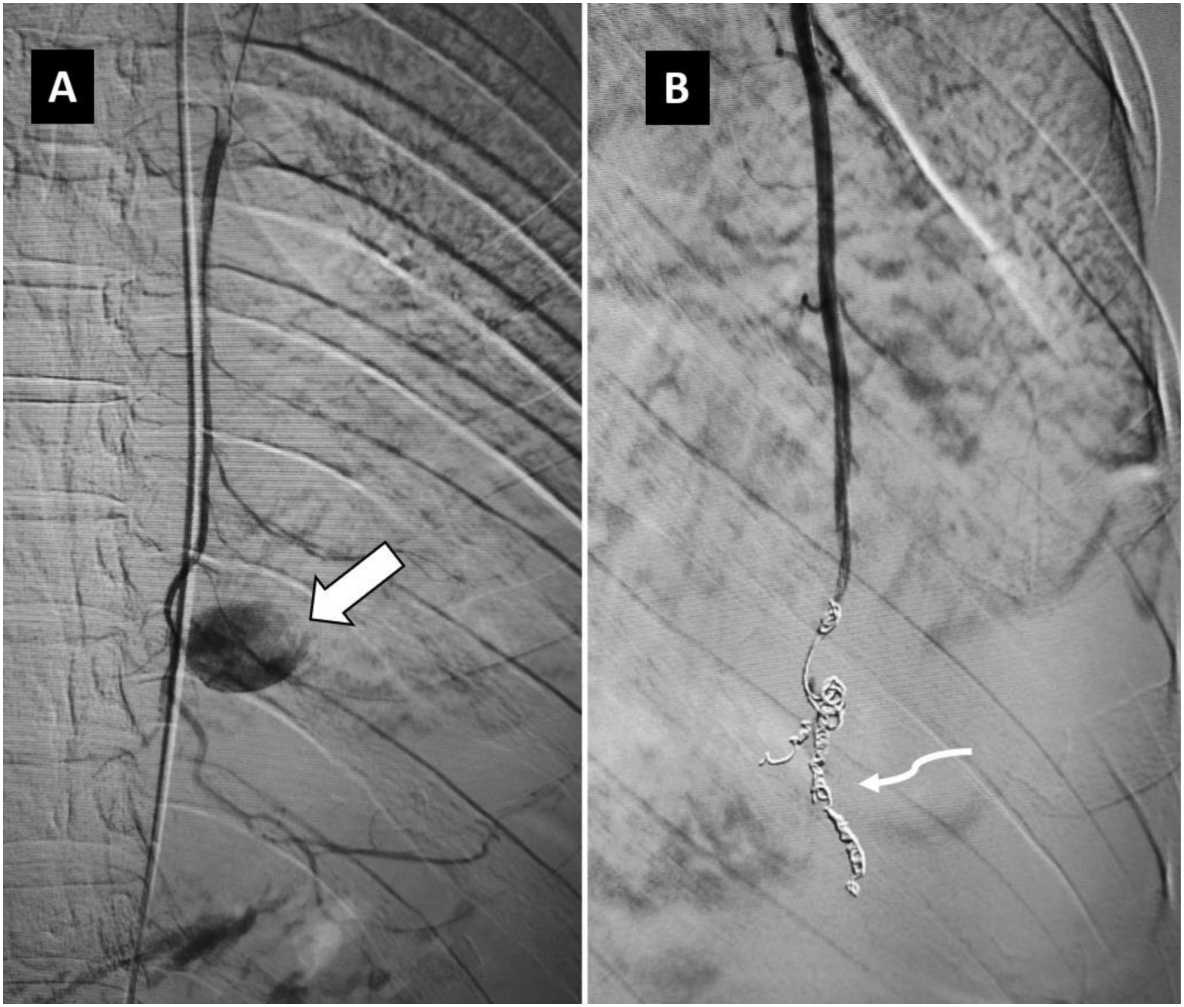
Digital subtraction angiography images following selective cannulation of the left internal mammary artery, revealing a contrast-filled outpouching arising from the artery (the solid arrow, picture A). Coil embolization (the curved arrow) was subsequently performed (picture B), with complete non-opacification of the pseudoaneurysm

By using right common femoral arterial access, superselective cannulation of the left IMA was performed with a microcatheter (*2.3F Progreat, Terumo Medical Corporation*, New Jersey, USA). The initial angiogram confirmed the presence of a pseudoaneurysm originating from the distal IMA, without active contrast-extravasation. The pseudoaneurysm was embolized by using the ‘sandwich technique’ with two 5 mm x 14 cm and two 4 mm x 14 cm *Nester* microcoils (*Cook Medical*, Bloomington, USA). Post-embolization, there was non-visualisation of pseudoaneurysm (Figure 2). The total procedure time was approximately 22 minutes.

The bilateral hemothoraces were drained by using 10F pigtail catheters. The patient’s dyspnea improved, vital signs stabilized, and hemoglobin levels returned to normal. The patient was discharged on the third day post-procedure with stable vitals.

**Figure 3:**
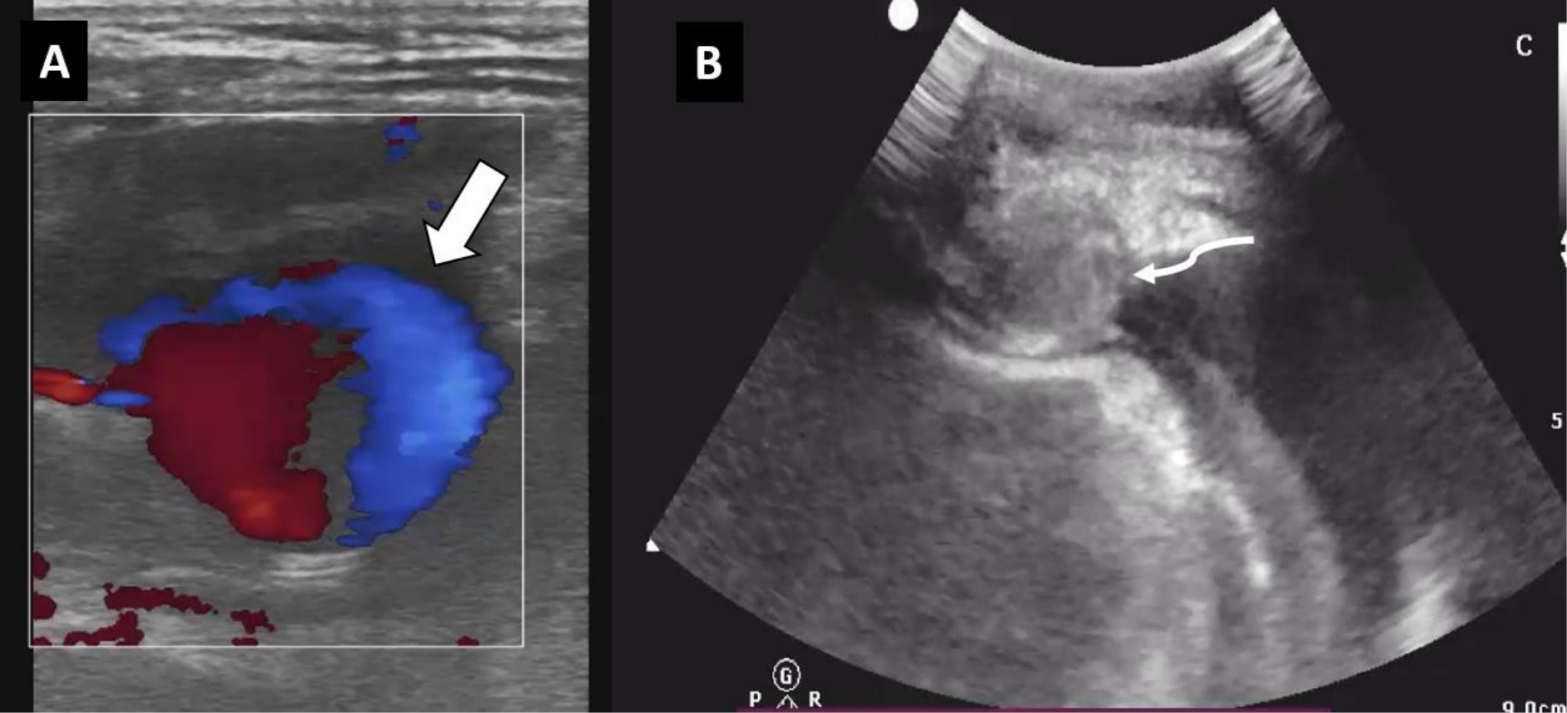
Pre-procedural USG (A) showing ying-yang phenomena on color doppler, suggesting pseudoaneurysm. Post-procedural USG (B) showing echogenic contents completely filling the previous pseudoaneurysm and suggesting complete thrombosis

A follow-up ultrasound one week later showed the outpouching, which initially had displayed the “ying-yang” phenomenon at presentation (Figure 3A), now showing echogenic contents completing filling it (Figure 3B), thus indicating complete thrombosis of the pseudoaneurysm. Follow-up echocardiograms at 1 week and 2 weeks post-procedure were normal without any hemopericardium.

## Discussion

IMA is a branch of subclavian artery. It descends 1–2 centimeters from the sternal margin, situated between the transversus thoracis muscle posteriorly and the internal intercostal muscles and costal cartilages anteriorly. [3] Pseudoaneurysm of IMA is a rare but potentially fatal case scenario.

Due to its anatomical trajectory, the internal mammary artery is inherently susceptible to traumatic injury, particularly from abrupt deceleration forces or penetrating trauma. Moreover, the thoracic cavity offers minimal supportive tissue for protection, while being in close proximity to the dynamic and constant motion of the chest wall. These anatomical and mechanical factors synergistically contribute for the formation of an IMA pseudoaneurysm. [4-6]

The reported causes of IMA pseudoaneurysms include sternotomy, vascular access procedures, trauma, fibromuscular dysplasia, polyarteritis nodosa, Kawasaki disease, systemic lupus erythematosus, Marfan syndrome, Ehlers-Danlos syndrome, type 1 neurofibromatosis, and chest wall infections caused by staphylococci, actinomycosis, tuberculosis, or fungi. [4,7-15]

Patients with an IMA pseudoaneurysm typically present with acute chest pain, shortness of breath, localized pulsatile swelling, hematoma, and skin changes. In addition to the pseudoaneurysm, these patients may also have associated conditions such as hemothorax, hemopericardium, or rib fractures. [4-7] It may further lead to life threatening complications such as pericardial tamponade, hemopneumothorax, diaphragmatic palsy, and hemorrhagic shock. [4-7]

In cases of traumatic pseudoaneurysms, the initial focus should be on stabilizing the patient’s vital signs, including the blood pressure, oxygen saturation, pulse, and respiratory rate. In certain specialized scenarios, additional interventions may be required, such as the insertion of an intercostal chest tube for pneumothorax or massive hemothorax, or performing pericardiocentesis for cardiac tamponade, as was necessary in our case. Historically, prior to the development of interventional radiology, treatment often involved sternotomy. However, open repair or sternotomy is typically associated with higher risks of tissue disruption, cardiopulmonary complications, infection, significant blood loss, and prolonged hospital stays.[6,7,16,17]

The management of pseudoaneurysms is primarily centered on individualized, targeted interventions, with an emphasis on minimizing invasiveness while optimizing clinical outcomes. Embolization should be performed to block the entry and exit of the neck of sac (the sandwich technique) as there can be distal collateral supply to IMA from epigastric arteries. Alternatively, glue embolization can be done in place of coils. This endovascular approach offers substantial advantages, including reduced recovery times and a lower incidence of postoperative complications.[4-7,10, 16,18-21]

In a case report by Kim et al., [11] the cause of the IMA pseudoaneurysm was neurofibromatosis, and it was managed successfully with endovascular embolization by using interlock coils. The pseudoaneurysm in the case discussed by Kim et al. was smaller (1.1 cm x 1 cm) compared to ours (1.5 cm x 2.1 cm x 2.2 cm).

Due to its superficial location, the simplest interventional approach for managing superficial pseudoaneurysms, such as those involving IMA, may be ultrasound-guided thrombin injection, as demonstrated by J. Jefferson et al. [20]

In another report by Yadav et al., [14] the IMA pseudoaneurysm resulted from tubercular empyema and was also managed with endovascular coil embolization. Unfortunately, the patient went into refractory shock following the procedure and could not be revived. The pseudoaneurysm in this case was larger (45 mm x 35 mm x 32 mm) than ours. Wani et al. [15] also described a case of IMA pseudoaneurysm caused by actinomycosis, leading to rapid deterioration of the patient’s vital signs and death before surgical repair could be performed. This underscores the serious prognosis of IMA pseudoaneurysms and highlights the importance of accurate diagnosis and timely intervention.

In contrast to the minimally-invasive endovascular approach, Phan et al. [5] in 1998 reported an open surgical repair (anterolateral thoracotomy) for a ruptured aneurysm. The reason for not using an endovascular approach at that time likely stemmed from its relative novelty, limited locally available resources. More recently, in 2021, Noh et al. [17] demonstrated that open repair via median sternotomy was used for ruptured IMA aneurysms, aiming to immediately relieve tamponade by removing the substernal hematoma and ligating the ruptured IMA.

While technically demanding, transcatheter embolization is associated with a markedly lower risk of hemorrhage compared to the traditional surgical approaches, such as open repair. Furthermore, the advent of percutaneous interventions, including the deployment of stent-grafts, has significantly enhanced therapeutic outcomes, contributing to a considerable reduction in both morbidity and mortality rates associated with pseudoaneurysms. These minimally invasive procedures represent a paradigm shift in the management of pseudoaneurysms, offering a safer, more efficacious alternative to open surgical intervention, with faster recovery and a markedly reduced complication profile, as demonstrated in our case. [4-7,10, 16,18]

## Conclusion

This case underscores the rare yet potentially fatal nature of IMA pseudoaneurysms, necessitating prompt diagnosis and early intervention. Stabilization of vital signs remains the foremost priority in such scenarios. CECT of the chest is instrumental in precisely localizing the pseudoaneurysm and delineating its dimensions, thereby facilitating targeted treatment planning. Endovascular embolization emerges as a highly effective, rapid, and minimally invasive alternative to open surgical repair, obviating the considerable morbidity and risks typically associated with open surgical interventions.
